# Diagnosing Developmental Dyscalculia on the Basis of Reliable Single Case FMRI Methods: Promises and Limitations

**DOI:** 10.1371/journal.pone.0083722

**Published:** 2013-12-09

**Authors:** Philipp Johannes Dinkel, Klaus Willmes, Helga Krinzinger, Kerstin Konrad, Jan Willem Koten Jr

**Affiliations:** 1 Interdisciplinary Center of Clinical Research “BIOMAT”, University Hospital of the RWTH, Aachen, Germany; 2 Section Neuropsychology, Department of Neurology, University Hospital of the RWTH, Aachen, Germany; 3 Section Child Neuropsychology, Department of Child and Adolescent Psychiatry, University Hospital of the RWTH, Aachen, Germany; 4 Cognitive Neuroscience, Institute of Neuroscience and Medicine (INM-III), Research Centre Jülich, Jülich, Germany; Lyon Neuroscience Research Center, France

## Abstract

FMRI-studies are mostly based on a group study approach, either analyzing one group or comparing multiple groups, or on approaches that correlate brain activation with clinically relevant criteria or behavioral measures. In this study we investigate the potential of fMRI-techniques focusing on individual differences in brain activation within a test-retest reliability context. We employ a single-case analysis approach, which contrasts dyscalculic children with a control group of typically developing children. In a second step, a support-vector machine analysis and cluster analysis techniques served to investigate similarities in multivariate brain activation patterns. Children were confronted with a non-symbolic number comparison and a non-symbolic exact calculation task during fMRI acquisition. Conventional second level group comparison analysis only showed small differences around the angular gyrus bilaterally and the left parieto-occipital sulcus. Analyses based on single-case statistical procedures revealed that developmental dyscalculia is characterized by individual differences predominantly in visual processing areas. Dyscalculic children seemed to compensate for relative under-activation in the primary visual cortex through an upregulation in higher visual areas. However, overlap in deviant activation was low for the dyscalculic children, indicating that developmental dyscalculia is a disorder characterized by heterogeneous brain activation differences. Using support vector machine analysis and cluster analysis, we tried to group dyscalculic and typically developing children according to brain activation. Fronto-parietal systems seem to qualify for a distinction between the two groups. However, this was only effective when reliable brain activations of both tasks were employed simultaneously. Results suggest that deficits in number representation in the visual-parietal cortex get compensated for through finger related aspects of number representation in fronto-parietal cortex. We conclude that dyscalculic children show large individual differences in brain activation patterns. Nonetheless, the majority of dyscalculic children can be differentiated from controls employing brain activation patterns when appropriate methods are used.

## Introduction

During the last two decades, images of the working brain have led to great hopes with respect to the clinical applicability of fMRI. Usually clinical populations are tested against controls, or brain activities of mixed populations are correlated with an external disease related criterion. This approach has led to broad knowledge about the development of cognitive abilities as well as about intervening factors. Analysis techniques focusing on the individual neural basis of behavioral disorders may support the diagnosis of a disorder. Additionally, such techniques may provide additional information about potential subgroups within a clinical population or, in a second step, even about the relation of a diseased individual to these subgroups. Focusing on developmental dyscalculia, we will show that these techniques do provide additional relevant information when standards of clinical research are applied to fMRI. 

Developmental dyscalculia (DD) is a specific learning disability in mathematics. According to the DSM-IV-definition the respective child’s mathematical ability is substantially below what one would expect considering age, intelligence and education, and it materially impedes academic achievement or daily living. It may possibly result from an impairment within particular parts of the brain involved in mathematical cognition (including language related brain circuits as well as areas of visual processing in particular in the parietal cortex) [1]. However, so far, DD cannot yet be identified based on the direct observation of brain functions, but has to be diagnosed based on tests of mathematical abilities in relationship to the child’s general IQ. This is difficult since there are many reasons (other than DD) for being bad at math, such as inadequate instructions, lack of motivation, attentional disorders, math anxiety, or across the board academic difficulties [1]. This illustrates the need to improve and adapt brain imaging techniques for diagnostic and clinical purposes.

 Twin studies and single gene studies suggest that developmental dyscalculia is a disorder of genetic origin [1,2]. The true targets of these genes in the brain remain elusive. It has been suggested that DD is related to a core-deficit in the ability to enumerate dots or to compare dots or Arabic numerals, the so-called number sense [3,4]. However, results from imaging studies seem to be characterized by divergent findings at the group level. Reports from comparable experiments range from no differences at all compared to typically developing children (TD) [5] to a wide range of brain areas that might possibly go along with the presence of DD [5–11]. Candidate brain areas include parietal areas, such as the intraparietal sulcus (IPS) and the posterior superior parietal lobe (PSPL), which have been related to number processing, but also visual and motor areas [5–12]. The partly visual nature of arithmetic and the differential visual processing of children with DD found in imaging studies suggest that cortical structures related to vision might be linked to the disorder.

The question remains, why findings are inconsistent. First, sample sizes used so far are acceptable for experimental fMRI studies. However, larger sample sizes could provide findings that are more representative for the disorder. Second, children presenting with DD differ in age, gender, developmental status, education, general intelligence, socioeconomic status, severity of the disorder and many more aspects influencing behavior. Hence, manifestation of DD is a heterogeneous phenomenon leading to inconsistent findings in fMRI activation studies. Similar heterogeneity has been found for many other developmental disorders, e.g. ADHD [13]. Therefore the question remains, whether group studies comprising just one undifferentiated sample of DD children are the appropriate method to get to the pathomechanisms underlying the disorder DD, or whether single-subject based analysis approaches should be pursued.

Within the context of single-case analyses, comparing an individual patient with some (healthy) control group, three methodological aspects seem to be relevant:

(1) Sufficient test-retest reliability of processed data; (2) sufficient comparability of affected individuals with a control group, calling for homogeneity within the control group; and (3) sufficient number and quality of clinically valid observations.

So far, no consensus exists with regard to standards for the reliability of fMRI activation contrast data. No agreed-upon criterion for fMRI studies is at hand. Within the fMRI literature, different minimum values for the evaluation of reliability by means of intraclass correlation coefficients (ICC, [14]) are discussed. Suggestions range from 0.4 [15] up to 0.5 [16]. ICCs between 0.4 and 0.6 have been considered as “fair” for univariate measures [17,18]. In most adult fMRI studies, results are in the 0.33 - 0.66 reliability range, when studies used ICC as an index of reliability [19]. By contrast, ICCs of fMRI results reported in child studies usually are below 0.33 [20]. This level of reliability is not sufficient for clinical purposes, but some suggestions have been made for improvement, such as increasing the number of observations and optimizing the fMRI-task design at the level of image acquisition as well as improving data analysis techniques at the level of preprocessing [19].

There are different possibilities to increase the number of observations. One can increase the number of volumes by means of parallel imaging techniques, by means of task length or by means of number of sessions. Recently, we showed that the reliability of child fMRI imaging could be improved using so-called “self-paced” task designs [21]. Whereas standard fixed-pace task designs use fixed stimulus length and inter-stimulus interval (ISI), self-paced task designs follow the performance speed of the individual child and thereby avoid both possible frustration and/or boredom by adjusting difficulty to ability levels as well as by preventing mind-wandering by assuring 100% time-on-task. Self-paced stimulus presentation may also be important for clinical populations, because large variability of performance speed is expected for varying levels of impairment. 

 At the level of preprocessing, there are several ways to improve data analysis techniques for better image quality. A promising method to increase reliability is a removal of variance due to head motion during image acquisition within the general linear model (GLM) framework [22]. The latter is of importance in studies where excessive head movement is almost inevitable such as in studies with children or patients suffering from ADHD, Parkinson’s or Huntington’s disease.

Sufficient comparability of individuals with and without a disorder can be attained by matching groups with respect to age, gender, educational level and broad performance measures such as general IQ. In addition, sufficient homogeneity within the control group is needed. Importantly, homogeneity at the level of behavior does not automatically imply homogeneity at the brain level. Individual persons with normal behavior may show unusual brain activation patterns, leading to higher variability within a control group as well. Heterogeneity of brain activation patterns in the control group may lead to low sensitivity of a diagnostic tool based on brain activation. As a consequence, homogenization of the control group can improve the quality of a clinical fMRI study aiming at the detection of some disorder.

The diagnosis of a disorder is usually based on a large number of diagnostic tests, which considered together lead to a clinical decision. Therefore it is important that a test is predictive for a disorder. Tests that are not predictive for a disorder are usually removed from a diagnostic battery. Applying these standards to fMRI, e.g. for the assessment of developmental dyscalculia, is challenging because one not only needs to know how to search but also where to search in the brain for the possible detection of different patterns of activation. 

Here we want to show how single-case methodology can be applied to fMRI activation data from dyscalculic children since individual classification is strongly required for clinical implementation. In a first step, established statistical test procedures for group analyses of fMRI data of children with and without DD are performed. 

In order to investigate whether potentially relevant brain regions identified in group studies are also suited for the individual diagnosis of DD, we will apply a statistical approach originally proposed for the single-case analysis of univariate behavioral data [23]. This approach was successfully applied to fMRI data recently [24,25]. The first study applied the approach to the (univariate) average activation level within a volume of interest, while the second study followed a massive voxelwise whole-brain approach.

In a third step, we will perform a support-vector analysis (SVA) as this has been established in clinical fMRI. 

In a last step, hierarchical cluster analyses using data of each paradigm separately and in a conjoint way will be carried out to study global similarities in extended brain activation patterns between individuals.

## Materials and Methods

### Ethics Statement

The study was approved by the local ethics committee of the Medical Faculty of the RWTH Aachen University and conducted according to the Declaration of Helsinki. Written and informed consent was obtained from the caregivers as well as orally from the children themselves.

### Participants

Out of a sample of 40 children (20 girls, 20 boys) in the age range from 6 years and 5 months to 10 years and 5 months with below average performance in diagnostic tests of number processing and calculation (below the 20th percentile for the total score of the dyscalculia test battery TEDI-MATH [26]), who participated in a larger training study, we selected all children who were diagnosed with developmental dyscalculia (at or below the 10^th^ percentile in the total TEDI-MATH score, estimated IQ-Score [27] above 85) and who did not move more than the equivalent of 1.5 voxels (5.25 mm) during the course of acquisition. The remaining sample (DD) consisted of 7 girls and 9 boys (n = 16). A control-group of typically developing children was also examined. The control-group was age-matched and tested for normal development of arithmetic skills by means of a respective test battery (MARKO-D [28]). Only children with a sufficient result in the test battery and very good to average grades in math in school were included in the control group. This control group (8 girls, 8 boys) was also part of a larger sample [29].

Mean age of the dyscalculic group (DD) was 8 years and 2 months (SD: 10 months), ranging from 7 years and 1 months to 9 years and 10 months. Estimated IQ [27] ranged from 88 to 117 (mean: 99, SD: 7). Mean age of the control group was 8 years and 2 months (SD: 11 months), ranging from 6 years and 8 months to 9 years and 7 months. Estimated IQ ranged from 93 to 147 (mean: 107, SD: 13). Dyscalculic children showed a significantly lower test result for the MARKO-D-test (TD: 51 (± 11), DD: 42 (± 10); t(29) = 2.35, p = 0.026). All children visited a regular primary school and were in the 1^st^ to 3^rd^ grade. No child of the dyscalculic or control group was diagnosed with ADHD or was prescribed any ADHD medication at the time of or prior to inclusion in the study. 

### Behavioral data recording and stimulus presentation

Before starting fMRI acquisition, children were instructed about the tasks and had to complete some practice trials outside the scanner. During fMRI acquisition, stimulus presentation and response recording were achieved using the software Presentation (Neurobehavioral Systems, Albany, CA, http://www.neurobs.com, accessed on 01/03/2013).

Children viewed the stimuli via MRI compatible video goggles (VisuaStimXGA, Resonance Technology) with a horizontal viewing angle of 30 degrees and a vertical viewing angle of 22.5 degrees. The virtual image corresponds to a 32 cm broad screen at 60 cm distance. Answers were given using a MRI compatible response box with four response buttons. The response box was placed centrally on the child’s belly and responses had to be given by pressing the leftmost button with the left index finger or the rightmost button with the right index finger respectively.

Because of the expected differences in reaction time between DD and TD, stimuli were presented in a self-paced stimulus design, which improves test-retest reliability for fMRI data [21]. Self-paced designs with a fixed number of stimuli inherently lead to an unequal number of time points between individuals. In the Information we show that the inherent individual differences in observed number of time points do not affect the quality of the imaging data within an experiment at the level of the individual (see Text S1, Figure S1-2). The non-symbolic comparison task was presented in four blocks, each consisting of six trials. The non-symbolic calculation task was presented in six blocks, each consisting of four trials. Each trial stimulus was shown until button press and children had no time restrictions to give their response. Between two trials there was a short interstimulus interval of 0.5 seconds. After each block, there was a resting baseline condition for 14 seconds. Phase jitter was implicitly introduced due to the self-paced character of stimulus presentation.

To further increase reliability, all paradigms were repeated in a second session (retest) on the same day. Children were taken out of the scanner to give them a short rest between the two scanning occasions.

### Tasks and stimuli

When we constructed the tasks, the following objectives were most important for us: First, children of very low number processing ability should be able to solve the tasks. Second, to minimize verbal production needed to solve the tasks in the scanner, the calculation task should consist of no carry addition problems. Third, the same numerical stimuli should be used for all tasks to make them comparable and allow for direct contrasts. Only the number range with numerosities from 2 to 5 and addition results from 5 to 9 meets the above defined criteria. Using such small numerosities, we are aware that we cannot be sure that children do not use subitizing. On the contrary, it is very likely that most children use a mix of subitizing, estimation and counting to solve the tasks. We believe that for children of this age range it is more ecologically valid to investigate processing of small numerosities than to try to force children to use one or the other strategy on any small numerosity between 3 and 5. So we opted for a solution with addition problems with no carry procedure (results < 10) but including stimuli in the subitizing range.

In the middle of the screen a fixation cross was presented during the rest condition. The same fixation cross was used to separate two circular disks that contained a variable number of dots ranging between 2 and 5 per disk. Black dots were presented on a white field. In half of the pairs, dots of both arrays had the same size, and in the other half, the overall area of dots was matched using a Matlab program developed by Dehaene and colleagues (available at http://www.unicog.org, accessed on 01/03/2013).

#### Non-symbolic comparison task

In this task, children had to press the right respectively left key with the corresponding index-finger, if the larger number of dots was presented at the right respectively left side of the screen. The number of dots presented on either side of the screen ranged from 2 to 5. The larger number of dots appeared on the left and right side of the screen with equal probability. 

#### Non-symbolic exact calculation task

The non-symbolic, exact calculation task required children to press a right-hand key with the right index finger if two simultaneously presented arrays of dots added up to 7, and a left-hand key with the left index finger if the two arrays added up to any other number. Addends ranged from 2 to 5 dots and results ranged from 5 to 9. The larger addend was equally often presented on the left and on the right side of the screen. Half of the problems had the result 7, the result of the other half was equally often smaller or larger than 7.

### MR acquisition

Imaging was performed on a 3T magnetic resonance scanner (Siemens Trio, Siemens Medical Systems, Erlangen, Germany) using a 12 channel head coil. To minimize head movement, children’s heads were comfortably stabilized with foam cushions. Functional images were obtained using an echo-planar image (EPI) sequence sensitive to blood oxygen level-dependent (BOLD) contrast with the following parameters: repetition time (TR) = 1.600 ms, echo time (TE) = 30ms, flip-angle (FA) = 72°, field of view (FOV) = 384 x 384, slice thickness (ST) = 3.5 mm with 10% gap, matrix size (MS) = 64 x 64, spatial resolution = 3.5 x 3.5 x 3.5 mm^3^, 30 axial slices parallel to the AC-PC line, PAT-mode = GRAPPA and acceleration factor PE = 2. A T1-weighted anatomical data set was obtained from each child after acquisition of the functional data (TR = 1.900 ms, TE = 2.52 ms, FA = 9°, FOV = 256 x 256, slice thickness (ST) = 1 mm, spatial resolution 0.98 x 0.98 x 1 mm^3^).

### Data processing

#### Preprocessing

BrainVoyager QX 2.2 (Brain Innovation, Maastricht, Netherlands, http://www.brainvoyager.com, accessed on 01/03/2013) as well as NeuroElf v0.9c (Jochen Weber, SCAN Unit, Columbia University, NYC, NY, USA, http://www.neuroelf.net, accessed on 01/03/2013) and Matlab R2011b (The MathWorks Inc., Natick, Massachusetts, USA, http://www.mathworks.com, accessed on 01/03/2013) were used for preprocessing and further data analyses.

#### Alignment of functional and structural data

Test and retest sessions were aligned separately. Four alignment steps were applied to the data of the test session: (1) Alignment of all functional volumes to the first volume of the last functional scan (temporally closest to the anatomical scans) executed with a motion correction procedure implemented in BrainVoyager QX (parameters: trilinear interpolation, full data set, maximum number of iterations = 100) (2). A two-step co-registration procedure was executed to align the first volume of the last functional scan to the structural scan in native space (3). Anterior as well as posterior commissure were defined manually as a starting point for an AC-PC-plane transformation using sinc interpolation (4). Reference points for the Talairach transformation were defined manually and the transformation was executed using sinc interpolation.

### Alignment information obtained in steps 3 and 4 was also applied to the functional dataset.

The alignment procedure for the retest session followed a different regime: Steps 1 & 2 were identical to the test session (3). The structural scan of the retest session was co-registered to the structural scan in AC-PC space of the test session. Alignment quality was controlled by visual inspection of each scan (4). Finally, Talairach coordinates from step 4 of the test session were used for the retest session as well.

#### Preprocessing of functional data

Slice scan time correction (scan order: ascending-interleaved 2; sinc interpolation), 3D motion correction, temporal high-pass-filtering (2 cycles) and spatial Gaussian smoothing (7 mm) were administered to the functional datasets. Preprocessed functional data were transformed into anatomical space and retransformed to a resolution of 3 x 3 x 3 mm^3^ using sinc interpolation. For transformation into standardized Talairach space, transformation data obtained by structural alignment was used.

#### Data analysis

Specific steps to improve reliability were carried out as follows. Data analysis included six steps. Estimation of first-level beta weights, reliability masking based on voxelwise ICC estimates, computation of standard second level group contrasts, comparison of individual DD children’s voxelwise fMRI activation data with the control group using Crawford et al.’s univariate test statistic for the detection of a deficit [23] in a massive univariate comparison approach, a ROI-based support-vector-machine analysis, and finally hierarchical clustering of individual children’s whole brain voxelwise fMRI data.

#### General linear model

To increase reliability, we carried out the following procedure. Contrast beta-values for each session (“activation minus baseline”) and task (non-symbolic magnitude comparison, non-symbolic exact calculation) for each individual child were estimated in a general linear model corrected for serial correlation, using a first-order auto-regressive model. The functional data were analyzed with a conventional block design using canonical hrf modulation. Motion parameters obtained from 3D motion correction were entered into the GLM as confounding covariates to remove this possible cause for noise. Beta weights were exported using NeuroElf. Subsequently, voxelwise beta-weights were averaged across test and retest session per task contrast to reduce measurement error. Finally, these averaged beta weights were analyzed with in-house software for Matlab and second level contrasts were computed via one sample t-tests. We have limited our analysis mainly to normal baseline contrasts that might have better diagnostic properties in a single subject imaging context due to sufficient reliability (See Text S1, Figure S3). 

#### Masking for reliability

First, we calculated one voxelwise reliability map for each task using the two-way random factors single measures intraclass correlation coefficient, ICC(2,1), quantifying the consistency of beta contrast estimates between the two sessions. In order to obtain a reliability mask, applicable on both DD and TD children, ICCs were estimated per voxel over all n = 32 children, using a modiﬁed script for computation of ICC coefficients (Arash Salarian; available at http://www.mathworks.com/matlabcentral/fileexchange/22099-intraclass-correlation-coefficient-icc, accessed on 01/03/2013). Finally, ICC maps for the two tasks (comparison and calculation) were averaged using Fisher’s z’-transformation to obtain one common reliability map for both tasks. As child studies usually show only poor reliability [20], a strict lower threshold for reliability was set. Only voxels with an ICC(2,1) > 0.33 (See Figure S4 for visualization) were considered in further data analyses (cf. 19). 

#### Standard second level group analysis

The averaged first-level GLM contrast beta weights were tested against zero for each task separately for each group with a threshold of p<0.01. Additionally, the two groups of children were compared directly for each of the two tasks with a threshold of p<0.01. Contrasts of beta-value maps were masked with the minimum ICC mask to increase reliability of activation results. Masked results were subsequently corrected for multiple comparisons with a Monte-Carlo cluster threshold estimation procedure at p<0.05 and visualized in BrainVoyager QX.

#### Single-case comparison analysis

Children in the DD group were compared individually with the whole control group using the voxelwise single-case t-statistic applied to the averaged beta-weights [23,25]. Single-case t-values were computed per voxel for each individual dyscalculic child, using a modified script from the NeuroElf toolbox. Maps were exported to a BrainVoyager QX-compatible format. From these individual maps, we calculated relative frequency maps, indicating the percentage of dyscalculic children with a significant (p<0.01) deviation from the control group for each task separately. Masked results were subsequently corrected for multiple comparisons with a Monte-Carlo cluster threshold estimation procedure at p<0.05 and visualized in BrainVoyager QX. For each task, we obtained two frequency-of-deviation maps: (1) DD vs. TD (activation) and (2) DD vs. TD (deactivation). 

#### Support vector analysis

A conventional multivariate pattern analysis approach was used that is based on linear support vector machines using the leave-one-out method. SVA was performed in two different ways. First, the whole multivariate set of voxelwise averaged beta weights within the ICC-mask was entered into the SVA for each task individually and simultaneously by concatenating the two contrast beta weight vectors of both tasks into one vector. In a second step, we performed the SVA based on regions of interest. To avoid circularity we selected 17 regions of interest that were defined in a previously published study using the same tasks in a larger sample of TD children [29]. Again, the averaged beta weights within the ICC-mask were used for the analysis. The power set of all subsets derived from the 17 ROIs (except for the empty set) was subjected subset-by-subset to the SVA. The SVA was run for each task individually and for both tasks concatenated into a common vector. This full power set approach is the classic and optimal approach but seldom used in neuroimaging because it is computationally expensive [29]. In order to maintain data quality, we did not consider applying different, maybe computationally less expensive, approaches.

#### Hierarchical cluster-analysis

The whole multivariate set of voxelwise averaged beta weights within the ICC-mask was entered into a hierarchical cluster analysis in two steps: First, children were clustered for each task individually; second, data of both tasks were analyzed simultaneously by concatenating the two contrast beta weight vectors of both tasks into one vector. The complete linkage criterion was used for clustering of the children, based on Spearman correlation coefficients computed over all voxels passing the ICC criterion. The rank correlation coefficient was chosen to capture monotone relationships among activation patterns and to be less sensitive to possible outlying activation values. Complete linkage was employed as a strict agglomeration criterion in order to obtain well separated clusters, if present in the data.

### Behavioral data

Reaction time (RT) and accuracy (ACC) were analyzed using Matlab. Test-retest reliability of reaction time was estimated using the two-way random factors single measures intraclass correlation coefficient, ICC(2,1), comparing consistency among the two sessions. Since fMRI data were based on averaged beta weights, a similar procedure for the behavioral data was used to keep methodological consistency with the imaging data. RT as well as ACC was averaged across both sessions. Behavioral data were tested for significant mean differences between both groups via a two-sample t-test. Due to the small sample size and the expected heterogeneity of data, also non-parametric Mann-Whitney-U-tests were employed. Pearson correlation coefficients were calculated to assess the relation between age and response time. Additionally, we performed the Crawford test for a deficit comparing each individual dyscalculic child with the control group.

## Results and Discussion

### Behavioral data

Neither parametric nor non-parametric tests revealed any significant difference in reaction time between both groups (Student’s t-test: numerosity comparison: t(30) = 0.97, p = 0.34; calculation: t(30) = 1.34, p = 0.19; Mann-Whitney-U: numerosity comparison: U = 107, n_1_ = n_2_ = 16, p = 0.44 two-tailed; calculation: U = 100, n_1_ = n_2_ = 16, p = 0.30 two-tailed). Dyscalculic children showed an average reaction time of 1427 ms (sd = 815 ms) and a median of 1245 ms (IQA = 292 ms) in the comparison task and an average of 5295 ms (sd = 3258 ms) and a median of 4749 ms (IQA = 842 ms) in the calculation task, respectively. The control group showed an average reaction time of 1212 ms (sd = 342 ms) and a median of 1142 ms (IQA = 409 ms) in the comparison task and an average of 4084 ms (sd = 1569 ms) and a median of 4065 ms (IQA = 2247 ms) in the calculation task, respectively. Even if the two groups presented with no significant differences in reaction time, we would like to point out that the children in the DD group are diagnosed with developmental dyscalculia on the basis of a much more elaborate test for dyscalculia. Reliability of the comparison task, estimated by means of ICC (2,1) was 0.94 and 0.85 for the calculation task, respectively. 

We estimated the correlations between age and response time for the whole group as such and separately within the two subsamples. For the whole group Pearson correlations of r = 0.25 (p = 0.17) and r = -0.14 (p = 0.45) were observed for the non-symbolic comparison and calculation task respectively. For the control group we observed correlations of r = -0.38, (p = 0.15) for the comparison task and r = -0.31 (p = 0.25) for the calculation task. For the DD group we observed a correlation of r = 0.57 (p = 0.02) for the comparison task. Visual inspection of the data plots revealed that this high correlation was due to one outlying individual child. After removal of this outlying data point from the analysis, we observed a correlation of r = 0.25 (p = 0.36, see Figure S5). For the calculation task correlation was r = -0.08 (p = 0.76). Thus, in our sample and task we could not find age-dependent effects on RT.

The deficit analysis revealed that only one child with DD showed a significantly longer reaction time for the comparison task (t(31) = 8.96; p<0.001). One other child with DD showed a significant difference for the calculation task (t(31) = 7.71; p<0.001). Note that the calculation task used in this study was very simple, allowing analysis of differences in brain activation patterns independent of task performance differences.

### fMRI data

Median ICC within the reliability mask was 0.43 (max = 0.91). Hence, overall activation contrast data quality was in agreement with fMRI reliability standards [17–19] and superior when compared to the usual ICC-range of other child studies [20]. Detailed information about the reliability level can be found in Table 1.

**Table 1:** Distribution of reliability-levels.

		**ICC-range**	**absolute**	**relative [%]**	
total voxels		-1 ≤ ICC(2,1) ≤ 1	106720	100	
excluded voxels		ICC(2,1) < 0.33	87641	82.1	
included voxels		0.33 ≤ ICC(2,1) ≤ 1	19079	17.9	100
	“poor”	0.33 ≤ ICC(2,1) < 0.4	6712		35.2
	“fair”	0.4 ≤ ICC(2,1) < 0.6	10801		56.6
	“good”	0.6 ≤ ICC(2,1) < 0.75	1540		8.1
	“excellent”	0.75 ≤ ICC(2,1)	26		0.1

**Figure 1:**
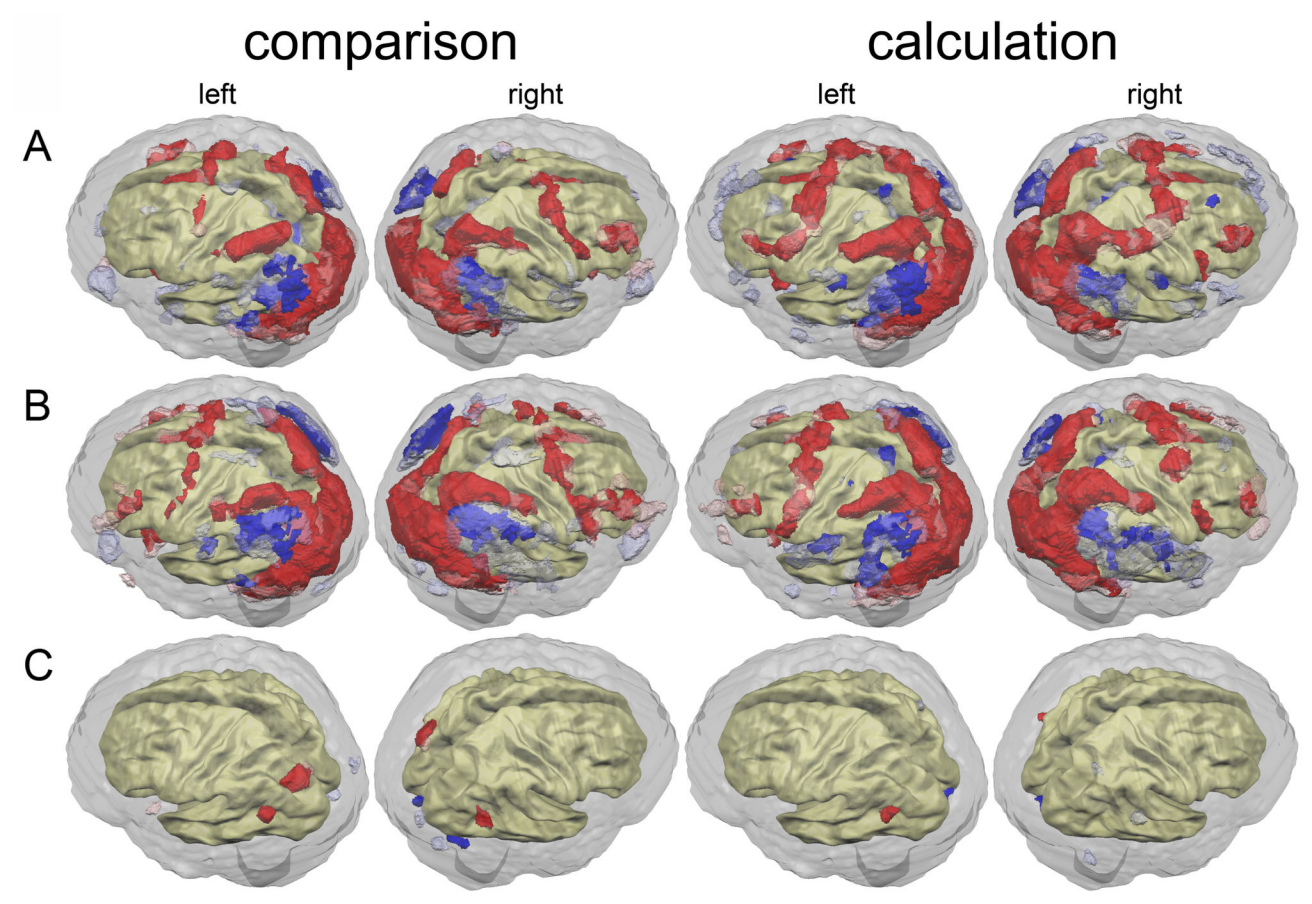
Results of standard second level group analyses. Reliable (ICC>0.33) and unreliable (ICC≤0.33) brain (de)activations (p<0.05, corrected) for the non-symbolic numerosity comparison task (left) and the non-symbolic exact calculation task (right). Red: Reliable activations; transparent red: unreliable activations; deactivations are analogously depicted in blue. A: Brain (de)activation of control children (TD). B: Brain (de)activation of dyscalculic children (DD). C: Brain activation differences between dyscalculic and control children. (Un-) reliable higher activations of the dyscalculic children are depicted in (transparent) red, lower activations are depicted in (transparent) blue.

Figure 1 shows that masking for reliability seems to be a highly effective method to denoise brain-activation patterns. Extended clusters of brain activation that do not carry reliable information are filtered out. But the mask also excludes potentially relevant activation aspects adjacent to important (possibly) function carrying structures such as right primary motor cortex in the calculation task.

Average brain activation patterns of DD and TD children appear to be quite similar for both tasks (cf. Figure 1C). Areas of differentiation between both groups are slightly more obvious for the comparison than for the calculation task.

In the non-symbolic comparison task, dyscalculic children appear to have more extended activations in left-hemispheric ventral premotor, inferior frontal and parieto-occipital cortex than control children. Moreover a small spot of activation around right Broca’s homologue was present in the dyscalculic group, while no such activation was found for the control group. An inferential comparison between both groups indeed showed a significant activation difference in the parieto-occipital sulcus that is in agreement with the previously observed activation pattern differences between groups. One significantly higher activated cluster was found in the left and right angular gyrus. In this case, both spots were less deactivated in dyscalculic children compared to the control group. Finally, a relative deactivation can be found in left primary visual cortex as well as in a part of the right cerebellum.

In the calculation task, brain activation patterns of the dyscalculic and control children appear to be almost identical. Slight differences were found around the left inferior frontal gyrus. The best obvious difference between the two groups was found for the pattern of deactivations. Dyscalculic children showed an extended area of deactivation in the right supramarginal region that was not present in the TD group. This difference in brain deactivation did not reach significance in the group contrast. In line with our observations for the comparison task, one significantly higher activated cluster was found in the left angular gyrus. Again, this difference can be explained by relatively less deactivation of this area in dyscalculic children. 

It has been suggested that the angular gyrus is activated in case of verbally mediated fact retrieval in arithmetic operations and processing of mathematical symbols [30–35]. A difference in deactivation level with relatively less activation for DD children is difficult to interpret. Alternatively, a less effectively deactivated angular gyrus might suggest that developmental dyscalculia is linked to a dysfunctional default network. The default network, or task-negative network, is a system that is usually deactivated during any task performance. It consists of the inferior parietal lobule (including the angular gyrus), hippocampal formation, temporal pole, medial prefrontal cortex as well as parts of the precuneus [36]. 

 An increase in activation around the parieto-occipital sulcus was previously described as indicating top-down regulation of spatial attention [37] that includes frontal eye fields and parts of parietal cortex. Again, only a small part of a larger network was found to be involved in dyscalculic children.

Our findings about differential activation patterns between dyscalculic and typically developing children only included parts of the default and attention network. Even though the networks as such are incomplete, the pattern of activations might have clinical relevance, if it can be found in the majority of dyscalculic children. We have investigated this aspect by comparing individual activation patterns of dyscalculic children with the group of typically developing children using the test for a deficit by Crawford and coworkers in a voxelwise fashion [23].

**Figure 2:**
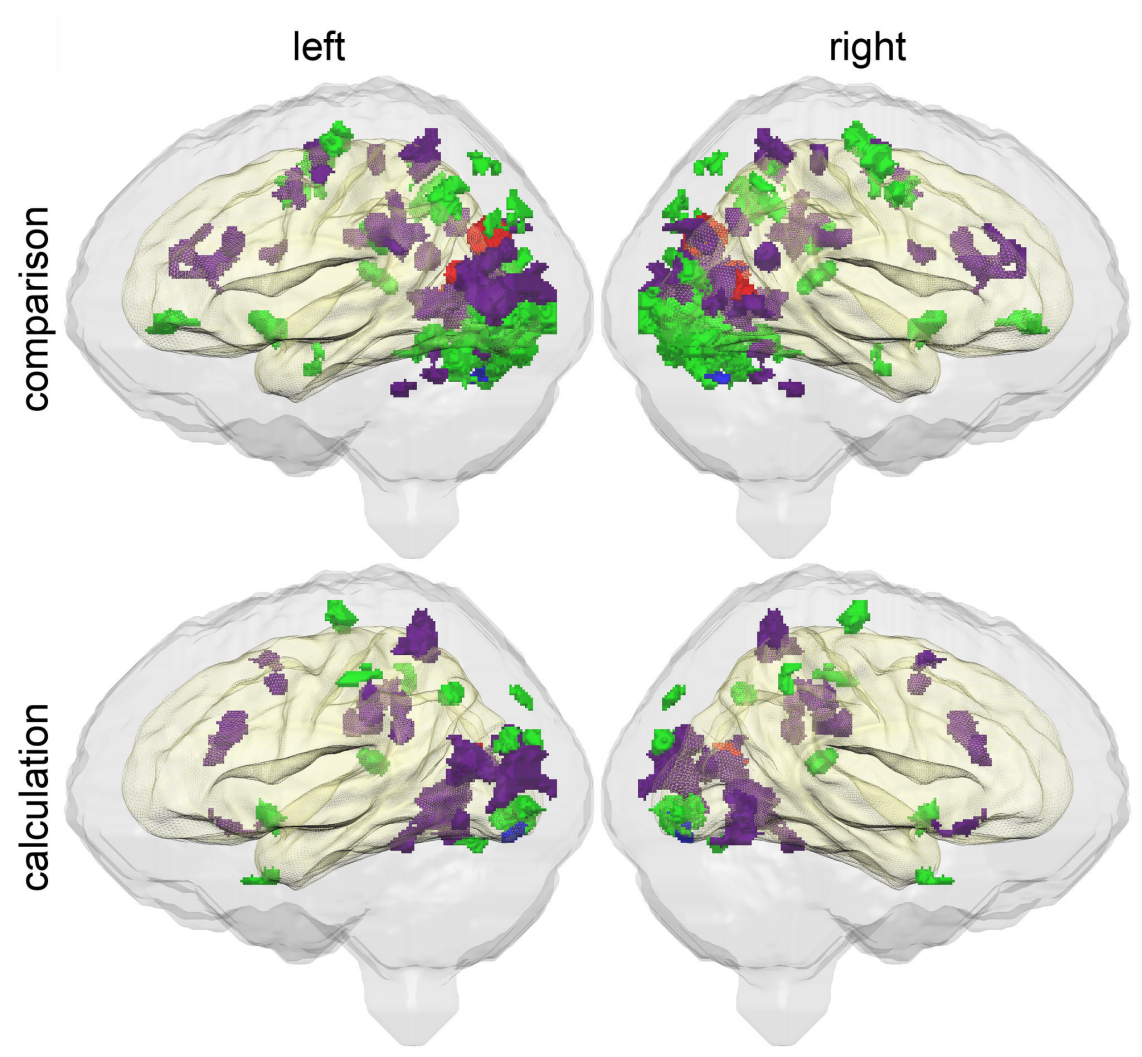
Overall extent of significant differences in brain activation pattern. Brain areas, where at least one dyscalculic child shows a significant difference (p<0.05, corrected) in brain activation in comparison to the control group as detected by means of the single-case comparison test by Crawford et al. [23] are conjointly visualized with areas that showed significant (p<0.05, corrected) between group differences as detected by the standard GLM (see Figure 1C). Relatively stronger or weaker activations as detected by means of the single-case comparison test by Crawford et al. [23] are shown in violet or green, respectively, whereas group effects follow the same color convention as in Figure 1C.

Figure 2 shows the group differences from Figure 1C as well as the results of the single-case comparison analysis. The overlap between the results of the two methods of analysis is rather limited compared to the overall extent of the significantly different activated areas. Activation differences traced by the general linear model may be due to an accumulation of small effects that possibly are not the essential difference regarding an individual child. Still parts of the brain activation differences detected by means of the GLM show overlap with individual brain activation differences. On the other hand, there are areas, which show a rather high frequency of individual brain activation differences not detected in the GLM group comparison. For the comparison task, the highest frequency of individual brain activation differences can be found in the cerebellar cortex (7 children, 43.75 %), parieto-occipital sulcus (5 children, 31.25 %) and the angular gyrus bilaterally (4 children, 25 %). For the calculation task, frequency of brain activation differences was rather low with a maximum in left angular gyrus (5 children, 31.25 %).

The overall qualitative impression from Figure 2 is an accumulation of cases that show under-activation in some part of the primary visual cortex and cerebellum and an accumulation of cases with an over-activation in higher visual systems. But from this visualization it remains unclear for how many children the latter holds true. 

**Figure 3:**
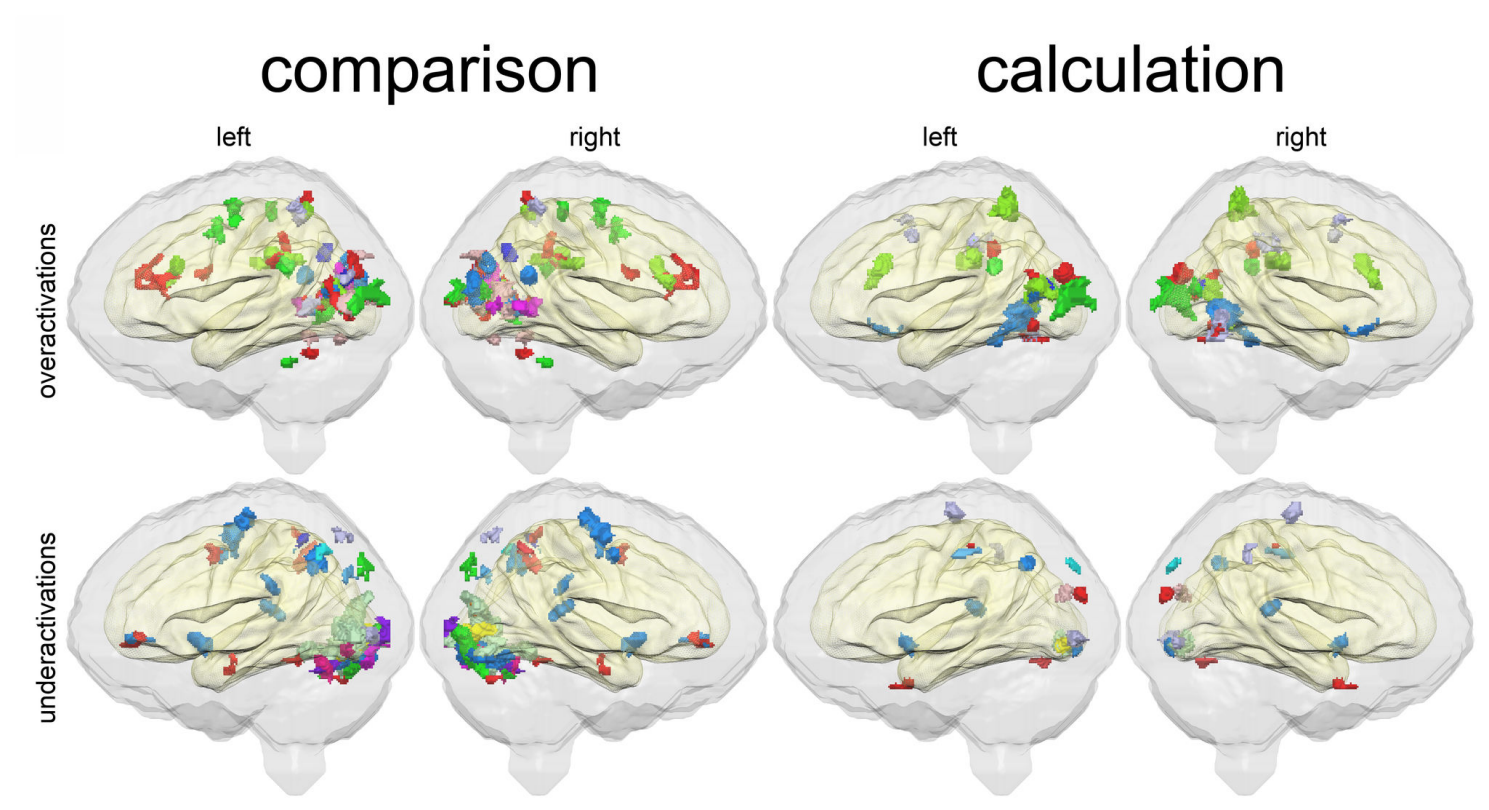
Significant differences in brain activation pattern for individual dyscalculic children. Brain areas where an individual dyscalculic child shows a significant (p<0.05, corrected) difference compared to the control group as detected by means of the single-case comparison test by Crawford et al. [23] visualized with a different color per child. Top row: relative over-activation for comparison (left) and calculation (right) task; Bottom row: relative under-activation for comparison (left) and calculation (right) task.

Informative with respect to the individual dyscalculic child is Figure 3. In this figure, the different dyscalculic children have different color codes. For the comparison task, 15 out of 16 children showed significant differences in brain activation when compared to the control group, while 11 out of 16 dyscalculic children showed significant differences in brain activation for the calculation task. These results clearly indicate that in the case of dyscalculia there are differences in brain activation patterns, but these differences are difficult to localize due to the heterogeneous distribution of brain activation differences. A high percentage of individual dyscalculic children showed atypical brain activation in some areas of the visual system. By contrast, frontal activation differences seem to play no major role in the disorder (at least for the tasks we used), because only 5 individuals out of 16 showed atypical frontal brain activation in at least one of the two tasks. The results of the single-case analysis using the approach of Crawford and coworkers suggest that diverging activations are diffusely localized in the parieto-occipital system and it seems difficult to characterize DD as a homogeneous entity. Neurofunctional heterogeneity of the disorder may explain the low consistency of brain activations reported in the various studies about DD, so far.

**Figure 4:**
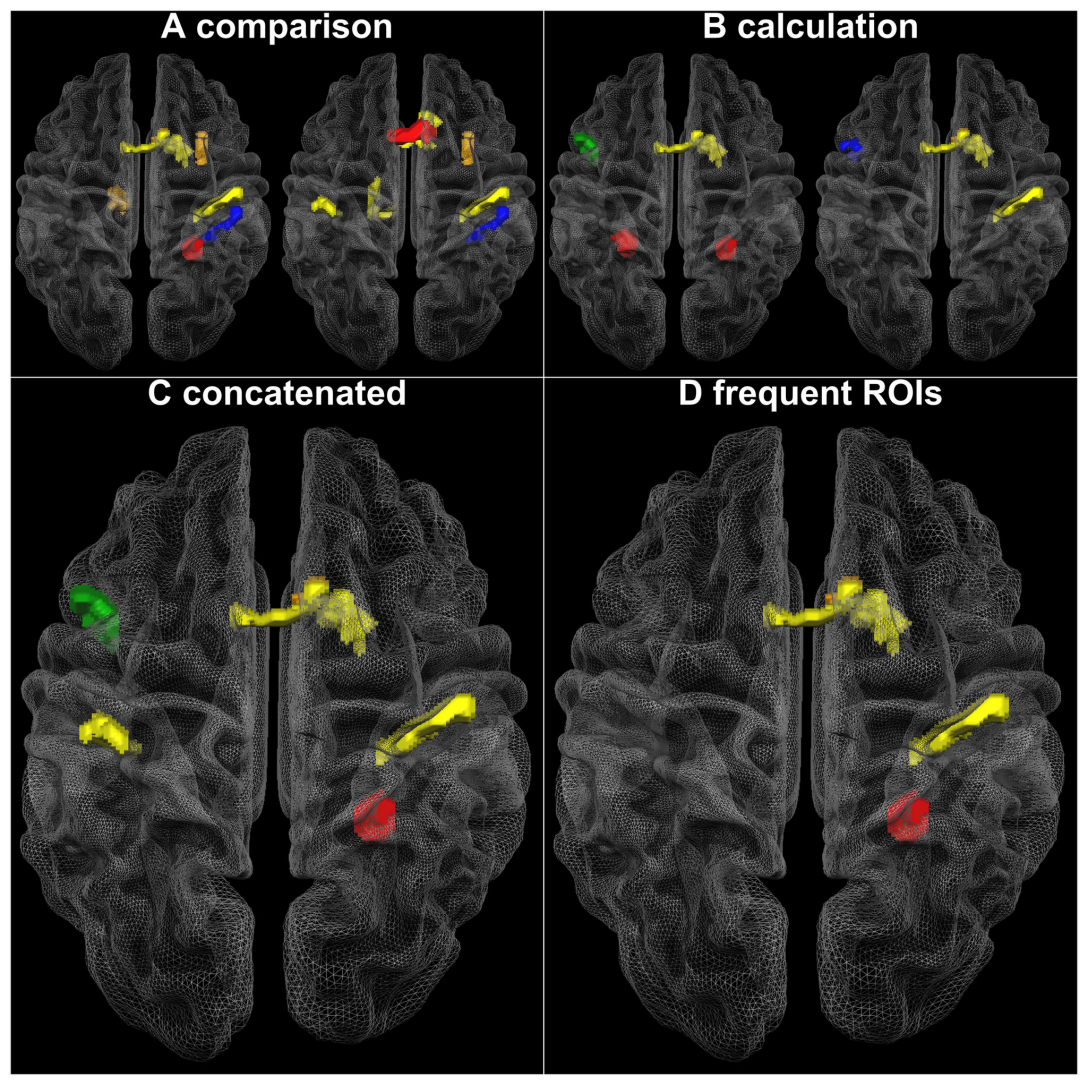
Combinations of ROIs leading to the best classification rate in the SVA. A. two combinations of 7 ROIs for the comparison task; B. two combinations of 4 ROIs for the calculation task, C. one combination of 6 ROIs for the concatenated vector, D. ROIs that led most frequently to the best classification.

Results from the whole-brain SVA showed a quite low correct classification rate (CCR) with 59%, 50% and 53% for the comparison, calculation and the concatenated vector, respectively. But, results from the ROI-based approach seem much more promising (Table 2). Here we report optimal classification rates found with the smallest number of features per task or task combination. For the comparison task a CCR of 87.5% was found with two different combinations of 7 ROIs, while for the calculation task a CCR of 81.25 % with two combinations of 4 ROIs was observed. The concatenated vector showed a CCR of 84.38 % with 6 ROIs (See Figure S6 for details regarding classification performance). The ROIs leading to these classifications differed from task to task, but in all cases involved some combination of frontoparietal areas (see Figure 4, Table 2). The right ventral and anterior intraparietal sulcus (vIPS resp. aIPS) as well as two aspects of the medial motor cortex occurred most frequently in the parsimonious classification solutions. In a previous study we showed that the aIPS and the medial aspects of the frontal cortex were associated with finger related aspects of number representation, while the vIPS is associated with poly-modal aspects of number representations in healthy children [29]. The possibility to differentiate between DD and TD regarding these regions could indicate a compensation of deficits in the polymodal aspects of number representation through finger related aspects of number representation in the DD group.

**Table 2:** Results of the support-vector analysis.

	**comparison**				**calculation**				**concatenated vector**	
**CCR [%]**	87.5				81.25				84.38	
**n**	7				4				6	
**Sensitivity [%]**	75		81.25		87.5		93.75		81.25	
**Specificity [%]**	100		93.75		75		68.75		87.5	
**ROIs**	THA	L	THA	L	vIPS	L	aIPS	R	vIPS	L
	vIPS	L	hIPS	R	vIPS	R	PMC	L	aIPS	R
	hIPS	L	aIPS	R	vPMC	L	PCL	R	aIPS	L
	aIPS	R	aIPS	L	aPCL	B	aPCL	B	vPMC	L
	PCL	R	CINS	B					PCL	R
	aPCL	B	CING	R					aPCL	B
	aFOP	R	aFOP	R						

*CCR*: maximum correct classification rate, *n*: number of ROIs needed for the best classification; *L*: left hemispheric; *R*: right hemispheric; *B*: bilateral; *THA*: Thalamus; *v/h/aIPS*: ventral/horizontal/anterior part of the intraparietal sulcus; *(a)PCL*: (anterior) paracentral lobule; *aFOP*: anterior part of the frontal operculum; *CINS*: cingulate sulcus; *CING*: cingulate gyrus; *vPMC*: ventral premotor cortex.

A final alternative analysis technique we used focused on the similarity of brain activation patterns instead of finding differences among individuals or groups. 

**Figure 5:**
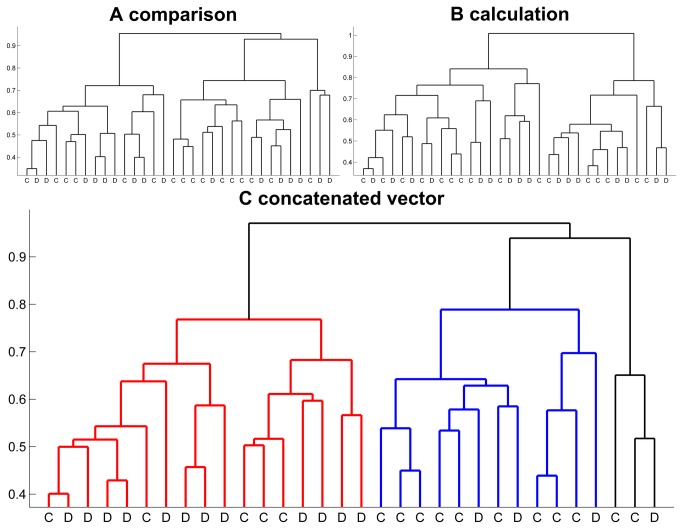
Results of hierarchical cluster analyses. Dendrograms of complete linkage hierarchical cluster analyses based on the vector of contrast beta weights per child within the reliability mask. D = dyscalculic child, C = control child. A. comparison task; B. calculation task; C. conjoint data of comparison and calculation task, cluster C1: red, cluster C2: blue, cluster C3: black.

Figure 5 shows the results of a complete linkage hierarchical cluster analysis for the whole multivariate pattern of contrast beta values of all n = 32 children within the reliability mask from the two tasks separately (Figure 5A-B) and in equally weighted concatenation (Figure 5C).

The comparison task (Figure 5A) revealed three clusters, one of them containing only 3 children. The remaining two clusters were almost identical in size (14 and 15 children). 6 children out of 15 in Cluster 1 and 9 out of 14 in Cluster 2 are control children. For the calculation task (Figure 5B) only two clusters were discerned. Cluster 1 contains 18 children (9 controls) and Cluster 2 contains 14 children (7 controls), indicating no between cluster differentiation of dyscalculic and typically developing children.

For the combined analysis of both tasks (Figure 5C), three clusters could be discerned, one of them again with only 3 children (C3). One large cluster (C1) has a majority of dyscalculic children, while the other cluster (C2) is dominated by control children (see Table 3). The two larger clusters show no significant difference in age or general intelligence. Detailed information concerning the characteristics of C1 and C2 is depicted in Table 3.

**Table 3:** Characteristics of Cluster 1 (C1) and Cluster 2 (C2).

		**C1**	**C2**	**t-statistic**
**N**		17	12	
**DD / TD**		12 / 5	3 / 9	
**sex (m / f)**		10 / 7	5 / 7	
**age (y)**	mean (± sd)	8.36 (± 0.71)	7.95 (± 0.81)	t(27) = 1.45, p = 0.16
	median (min; max)	8.41 (7.30; 9.81)	7.73 (6.87; 9.17)	
**IQ**	mean (± sd)	101 (± 14)	103 (± 7)*	t(26) = 0.57, p = 0.71
	median (min; max)	100 (88; 147)	103 (93; 117)	
TEDI-Math total score (PR)**	mean (± sd)	6.3 (± 3.5)	5.7 (± 5.1)	
	median (min; max)	7 (1; 10)	7 (0; 10)	

PR: percentile rank

^*^ one missing value of a typically developing child

^**^ TEDI-Math total score was only acquired from dyscalculic children

The improved separation of dyscalculic and typically developing children in the cluster analysis employing two paradigms poses the question whether the diagnostic quality of fMRI-driven cluster analyses can be improved by including several tasks or more specific tasks, like in diagnostic procedures based on behavioral tests.

**Figure 6:**
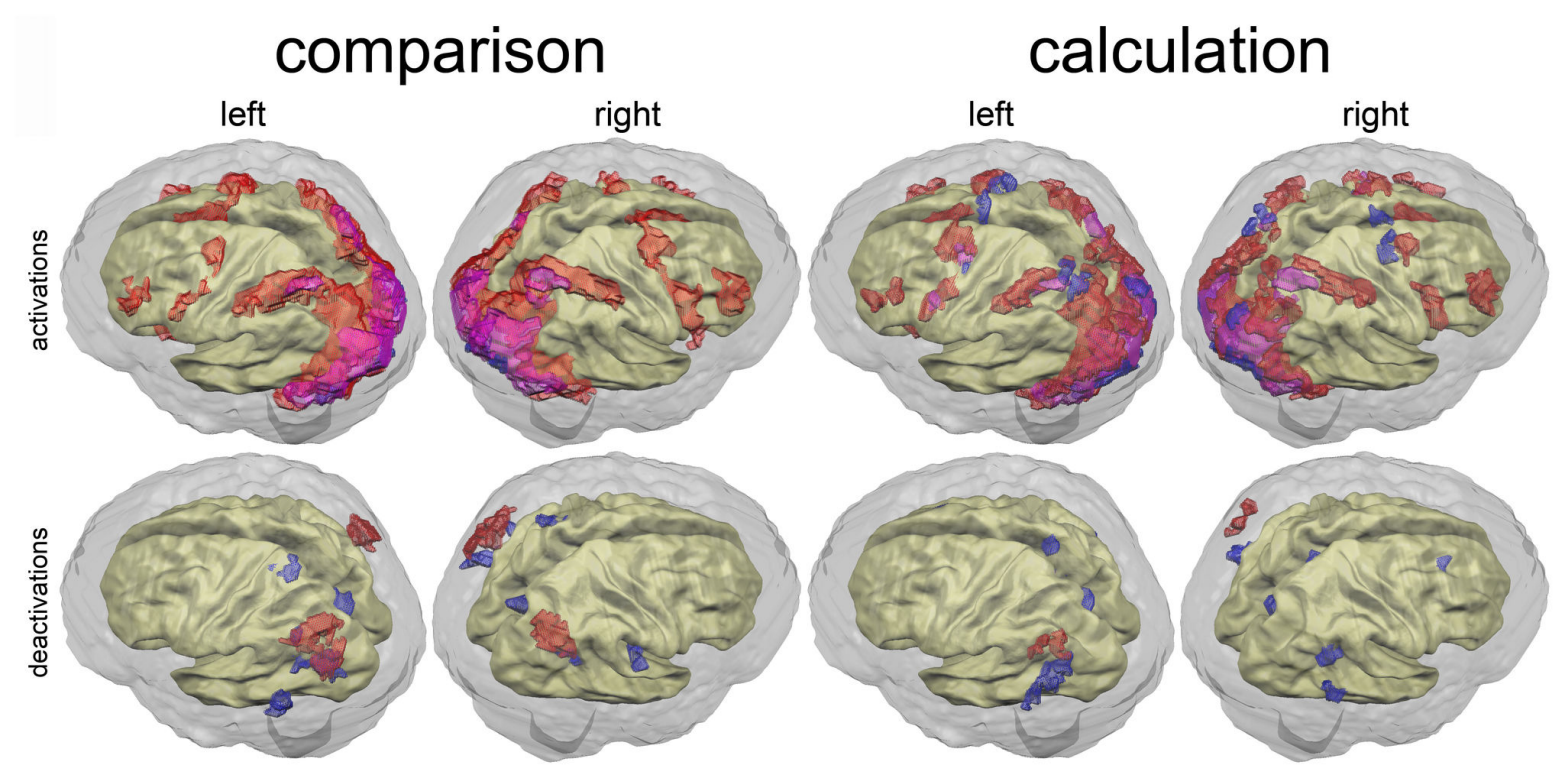
Second level group analysis of two clusters. Second level group analysis data tested against baseline for the two groups that were obtained from a conjoint cluster analysis (see Figure 4C). Brain (de)activations of the predominantly dyscalculic (C1) and the predominantly control (C2) cluster are depicted in red and blue respectively, overlap is depicted in pink.

Results of the combined analysis clearly show that complex developmental disorders like developmental dyscalculia are probably better diagnosed with a multivariate approach. The final question is what the pattern of brain activation of the two distinct clusters might be. Figure 6 visualizes second level group analyses for the children from the two major clusters using activation condition against baseline contrasts for each group. We did not compute direct contrasts between groups because prior group homogenization via cluster analysis would lead to circular results and to a false impression of between-group differences. To demonstrate the potential advantage of clustering techniques for future studies, baseline-contrasts were computed and depicted in Figure 6.

To a certain extent, the cluster analysis seems to synthesize results from the direct group contrasts as well as the single-case analysis. The similarity with the between group analysis is in particular visible for the higher activation of the parieto-occipital sulcus in the dyscalculic cluster (C1), as well as the inability of these children to deactivate the brain as inferred from areas of the default network. With respect to the single-case analysis, some similarities were found for the visual systems. Children in the dyscalculic cluster seem to show less extended activation in lower visual systems, but more extended activation in higher lateral and ventral visual systems. It is known that spatial frequency selective cells exit within the striate cortex that responds to the number of presented lines [38,39]. It is not entirely clear if these cells respond to dots as well but we speculate that spatial frequency coding deficits in the visual system might in some cases contribute to developmental dyscalculia. Thus, the non-symbolic comparison task might rely more on spatial frequency coding whereas the non-symbolic calculation task might rely more on a sequential search in the visual field. Within this context it is interesting to notice that the non-symbolic comparison task showed more signs of relative deactivation when compared to the non-symbolic calculation task. Thus the neural correlates of magnitude coding deficits might be related to developmental dyscalculia.

However, the cluster analysis and the ROI-based SVA also introduce another aspect that did not appear in the conventional direct group contrast or the single-case analysis. Namely a pronounced activation of the frontoparietal systems found for the dyscalculic cluster (C1) that is substantially less present for the control cluster (C2). These frontoparietal systems essentially contributed to the good classification rate found in the SVA. To this end, one might speculate that developmental dyscalculia is probably a disorder of lower visual systems that may require compensation through frontal top-down regulation of higher visual systems. We would like to point out that these discussions remain highly speculative, especially since both clusters comprise dyscalculic and typically developing children. However, our findings are in line with a recent study including children with and without ADHD that also demonstrated that typically developing children can be classified into distinct neuropsychological subgroups. This normal variation of typically developing children might also hold true for children with ADHD [40] or developmental dyscalculia, leading to a heterogeneous pattern of neuropsychological test results.

However, it was not so much the purpose of this article to isolate the potential neuropsychological correlates of developmental dyscalculia but to open a new window on the use of fMRI in clinical settings. Within this perspective, single-subject tests seem to be very relevant. Results of comparing individual DD children with the TD control group should be concordant with results of behavioral tests. Only when this type of validation of fMRI data by behavioral measures is obtained, fMRI might have implications for therapy evaluation. 

## Conclusion

In our study we introduced complementary analytic alternatives to the standard general linear model approach for fMRI activation data. These alternatives provide an opportunity to perform single-case analyses as well as relating individual brain activation patterns to those of a reference group. These promising approaches may not only help along the way towards understanding developmental disorders from a neural perspective but also towards understanding the success of treatment. Despite the promising results obtained in this study we would like to emphasize that fMRI based diagnostics is by no means as good as standard diagnostic tests that are based on behavioral measures only. However, fMRI test batteries that are based on a larger number of cognitive phenotypes might improve the predictive validity of fMRI based diagnostics. 

Our study detected deviating neural mechanisms in the dyscalculic group even though no differences in performance were observed. The single-case analysis revealed that dyscalculic children show a shift of activation from primary to higher visual systems. Additional analyses suggest that this shift goes along with higher activation in frontoparietal cortex which could represent a compensation of deficits in the polymodal aspects of number representation through finger related representation in the DD group. We argue that these differences in brain activation in the absence of behavioral differences can be interpreted as stable compensatory neural mechanisms that have evolved over time. For this reason the multivariate pattern approaches were able to differentiate the two groups, even if there were no differences in performance. The different brain areas detected through multivariate pattern analysis suggest that future connectivity analysis approaches might provide further insights in the neuroanatomical basis of developmental dyscalculia.

## Supporting Information

Figure S1 **Relation between first level reliability estimates and brain activation threshold.** This figure depicts the relation between the reliability estimate (top row: Dice overlap, bottom row: ICC) and the brain activation threshold at which the reliability was estimated for the non-symbolic number comparison task (left column) and the non-symbolic calculation task (right column) for all 32 individual children. On the vertical axis the reliability estimate is depicted, on the horizontal axis the p-value at which the contrast was thresholded. The mean reliability curve is depicted in bold black.
(TIF)

Figure S2 **Correlation between first level reliability estimates and reaction times at a given brain activation threshold.** The correlation (y-axis) between first level reliability estimates (see Figure S1) and reaction times of all 32 individual children at a given brain activation threshold of 0.05 > p > 0.00005 (x-axis).
(TIF)

Figure S3 **Effect of narrow contrasts on reliability.** A. Reliability map for the comparison task. B. Reliability map for the calculation task. C. Reliability map of the direct contrast calculation – comparison. D. t-statistics for the direct contrast calculation – comparison at a threshold of p < 0.01. Color code for A-C in leftmost column, for D in rightmost column.
(TIF)

Figure S4 **Reliability map for masking.** Reliability map used for masking the brain activation data of the standard second-level analysis (see Figure 1 in the Manuscript), obtained through a voxelwise averaging of the Fisher’s z’-transformated reliability estimates of both tasks.
(TIF)

Figure S5 **Relation between age (x-axis) and reaction time (y-Axis) of the dyscalculic group for the comparison task.** High (positive) correlation (r = 0.57, p = 0.02) was found due to the one outlier (right upper corner). After removal of the outlier from the analysis, no significant correlation (r = 0.25, p = 0.36) could be found for this group and task.
(TIF)

Figure S6 **Support-vector machine analysis.** This figure depicts the success of the support-vector machine analysis. Top: best correct classification rate (y-axis) for each number of ROIs (x-axis) for each condition. Bottom: Number of different combinations of ROIs (y-axis) that reached the best correct classification rate for each number of ROIs (y-axis). Blue: comparison task, red: calculation task, black: concatenated vector.
(TIF)

Text S1 **Further analyses concerning reliability in single-subject imaging.** a. Impact of block time differences due to the self-paced study design.
b. Influence of narrow contrasts in single-subject imaging.
(DOCX)
