# Supplementary figures and images for: Diagnosing Developmental Dyscalculia on the Basis of Reliable Single Case FMRI Methods: Promises and Limitations
Source: PLoS One. 2013 Dec 9;8(12):e83722. doi: 10.1371/journal.pone.0083722 (PMC3857322; doi:10.1371/journal.pone.0083722)

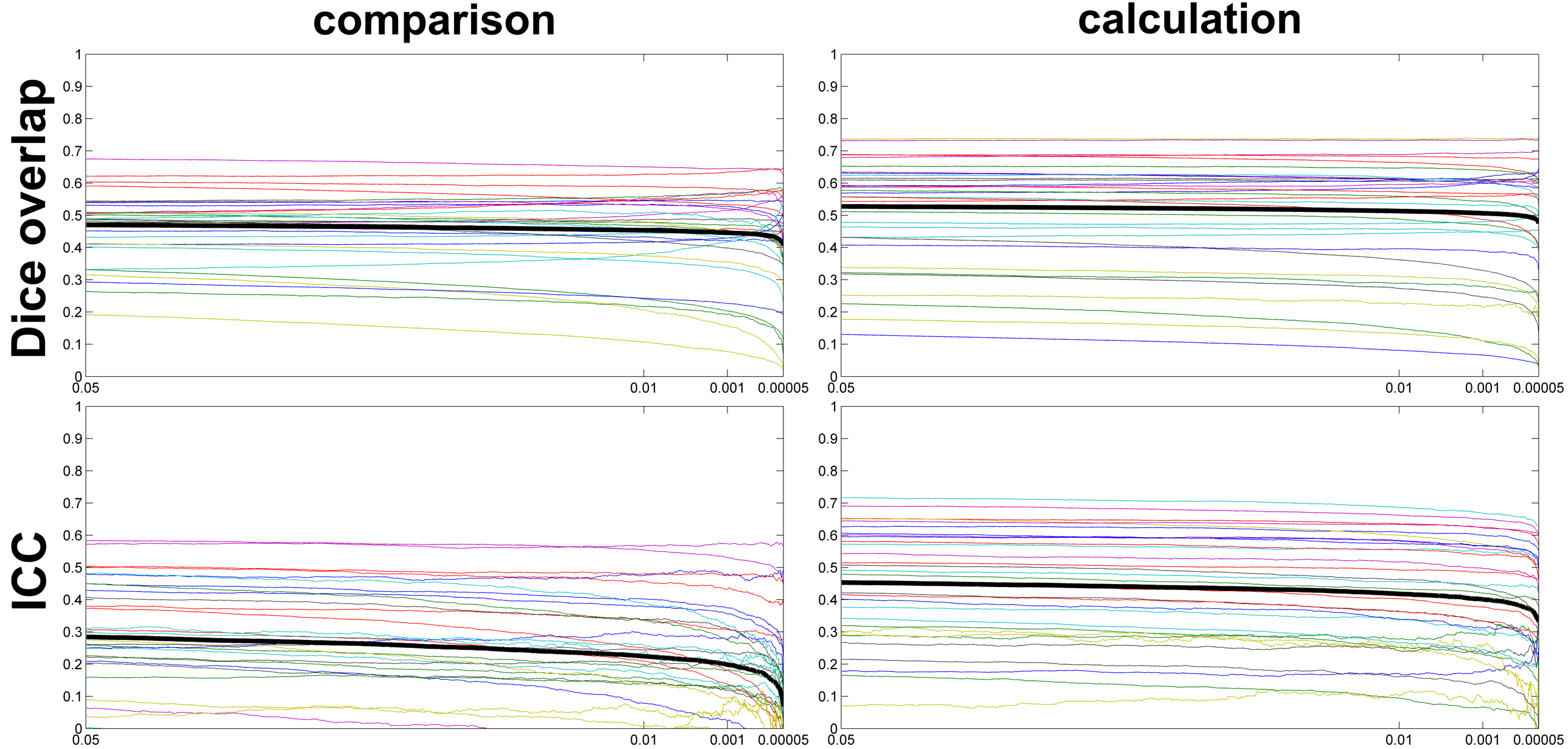

Supplement: Figure S1 — Relation between first level reliability estimates and brain activation threshold. This figure depicts the relation between the reliability estimate (top row: Dice overlap, bottom row: ICC) and the brain activation threshold at which the reliability was estimated for the non-symbolic number comparison task (left column) and the non-symbolic calculation task (right column) for all 32 individual children. On the vertical axis the reliability estimate is depicted, on the horizontal axis the p-value at which the contrast was thresholded. The mean reliability curve is depicted in bold black. (TIF) [file pone.0083722.s001.tif]

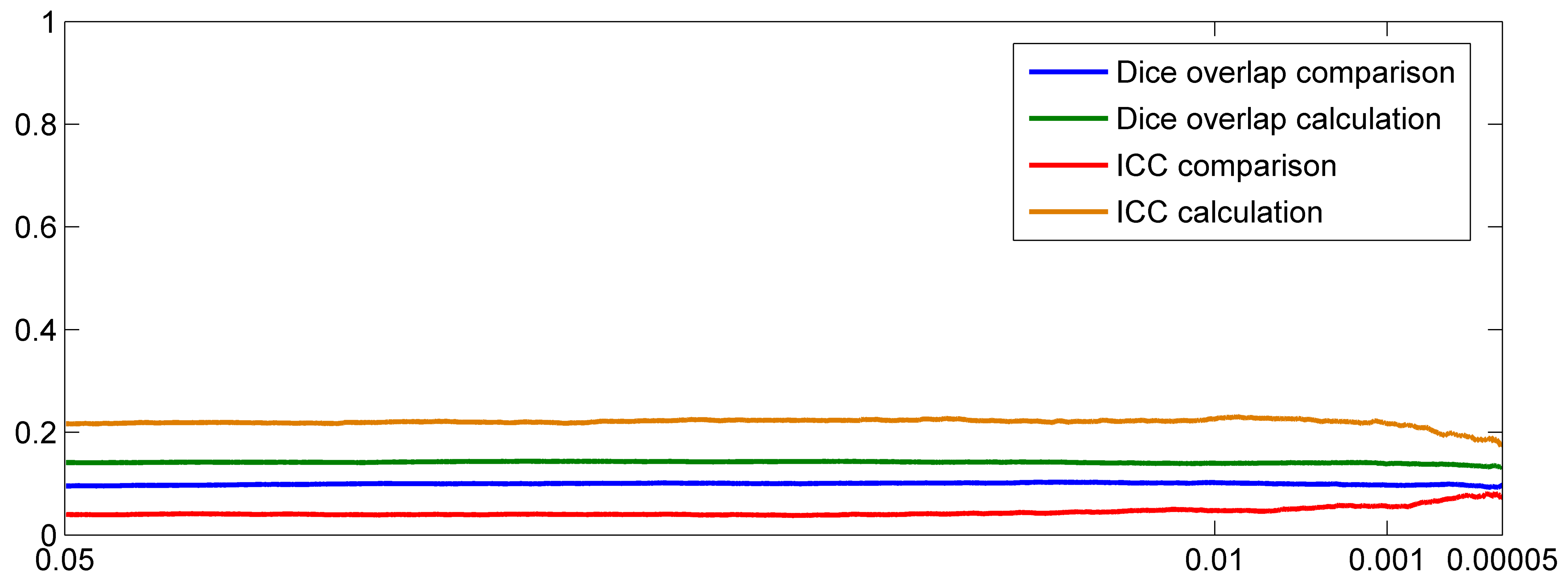

Supplement: Figure S2 — Correlation between first level reliability estimates and reaction times at a given brain activation threshold. The correlation (y-axis) between first level reliability estimates (see Figure S1) and reaction times of all 32 individual children at a given brain activation threshold of 0.05 > p > 0.00005 (x-axis). (TIF) [file pone.0083722.s002.tif]

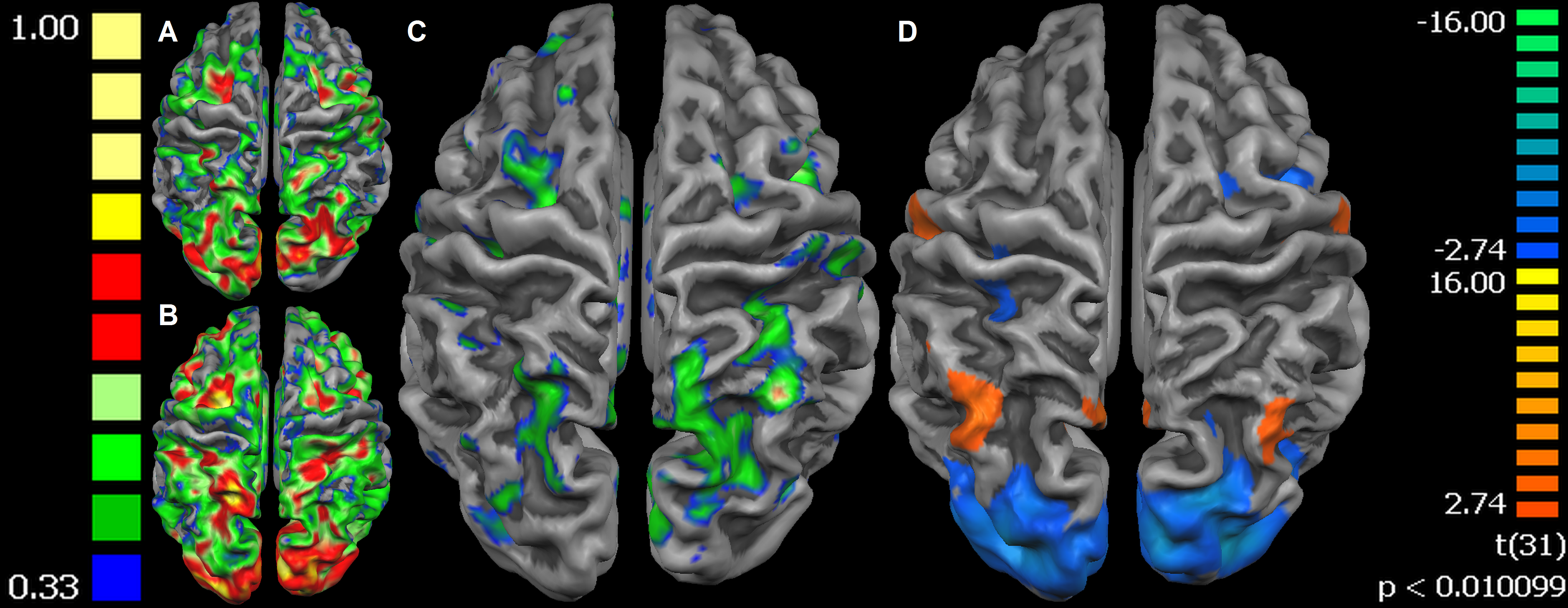

Supplement: Figure S3 — Effect of narrow contrasts on reliability. A. Reliability map for the comparison task. B. Reliability map for the calculation task. C. Reliability map of the direct contrast calculation – comparison. D. t-statistics for the direct contrast calculation – comparison at a threshold of p < 0.01. Color code for A-C in leftmost column, for D in rightmost column. (TIF) [file pone.0083722.s003.tif]

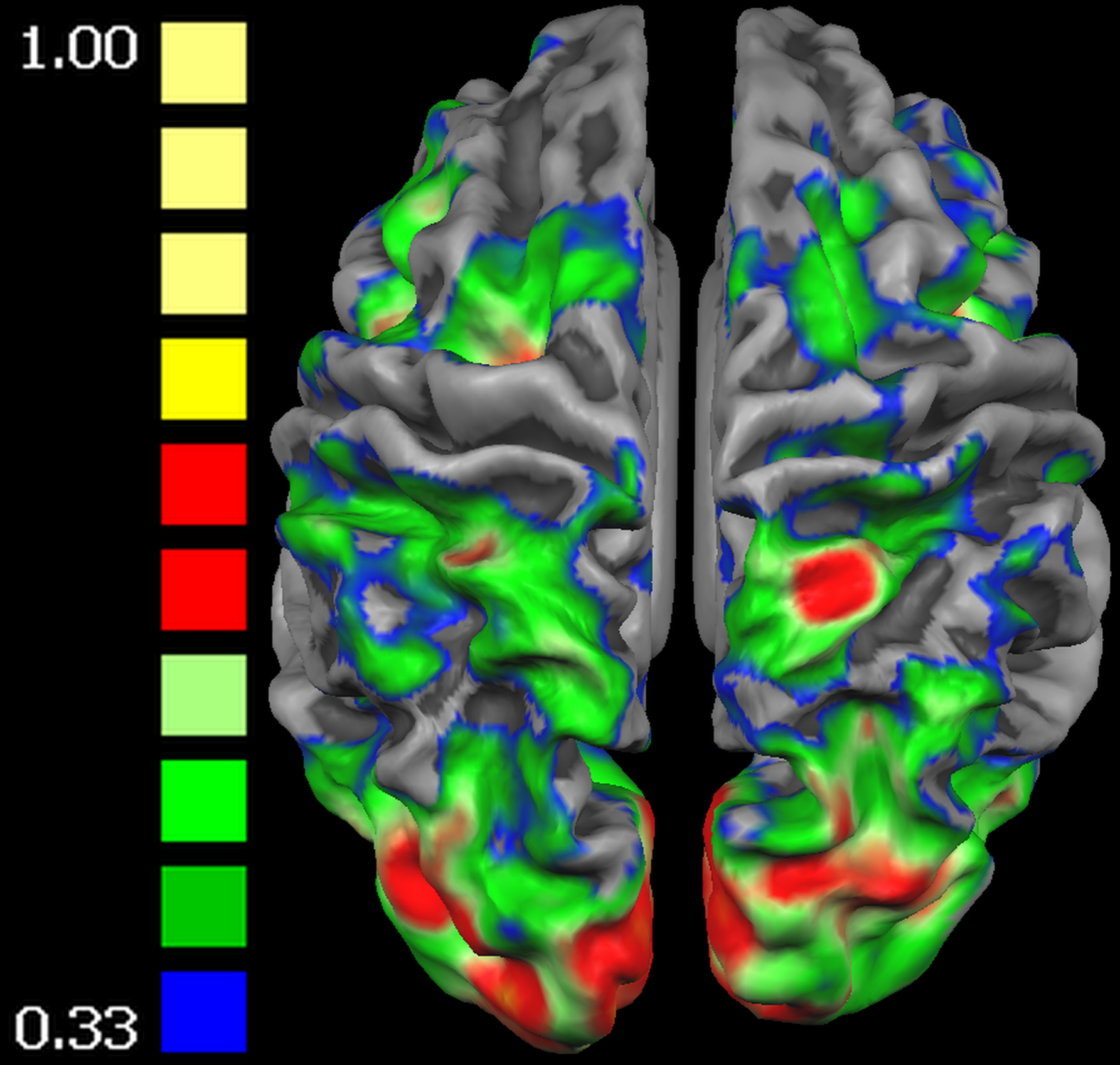

Supplement: Figure S4 — Reliability map for masking. Reliability map used for masking the brain activation data of the standard second-level analysis (see Figure 1 in the Manuscript), obtained through a voxelwise averaging of the Fisher’s z’-transformated reliability estimates of both tasks. (TIF) [file pone.0083722.s004.tif]

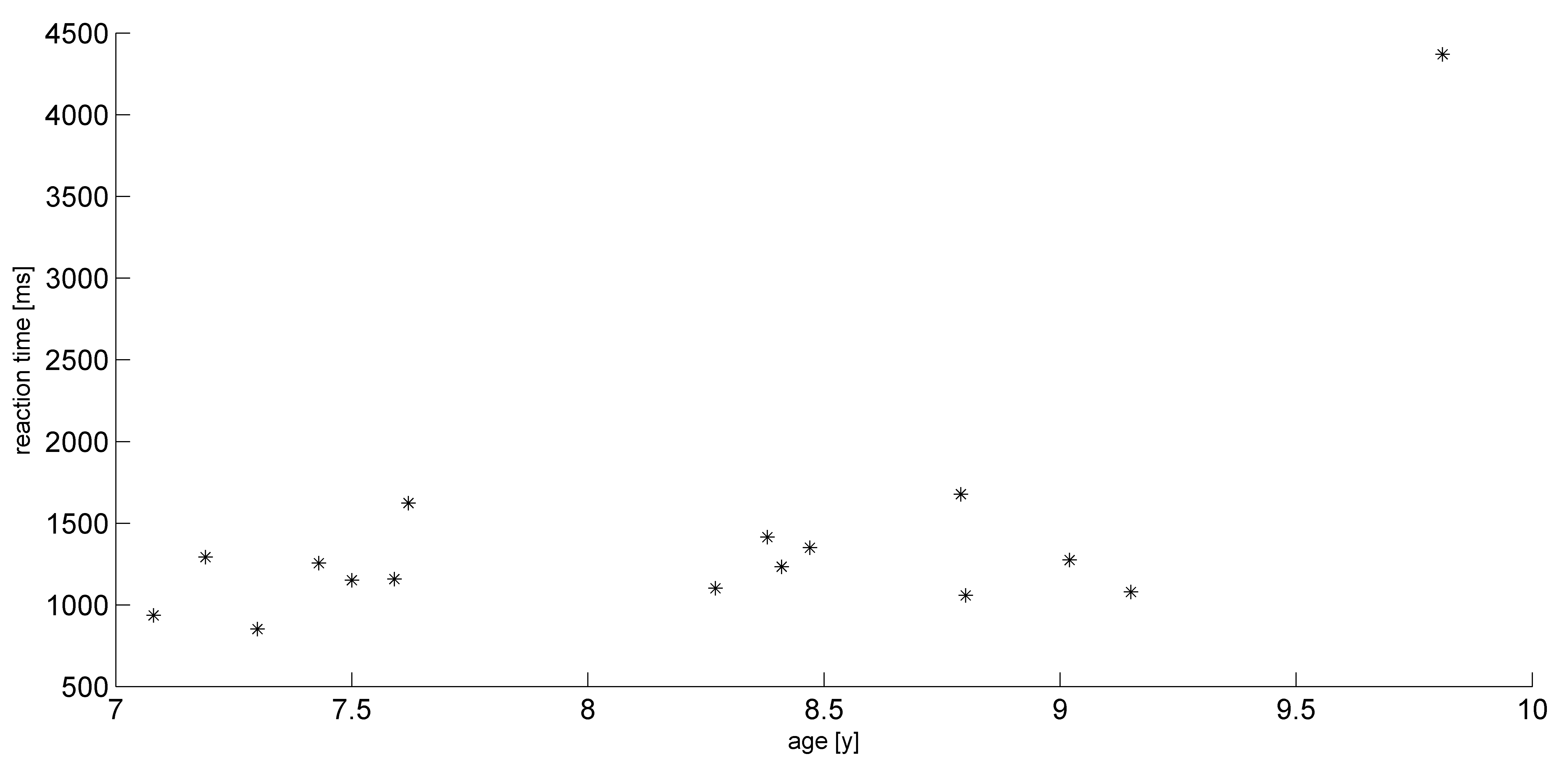

Supplement: Figure S5 — Relation between age (x-axis) and reaction time (y-Axis) of the dyscalculic group for the comparison task. High (positive) correlation (r = 0.57, p = 0.02) was found due to the one outlier (right upper corner). After removal of the outlier from the analysis, no significant correlation (r = 0.25, p = 0.36) could be found for this group and task. (TIF) [file pone.0083722.s005.tif]

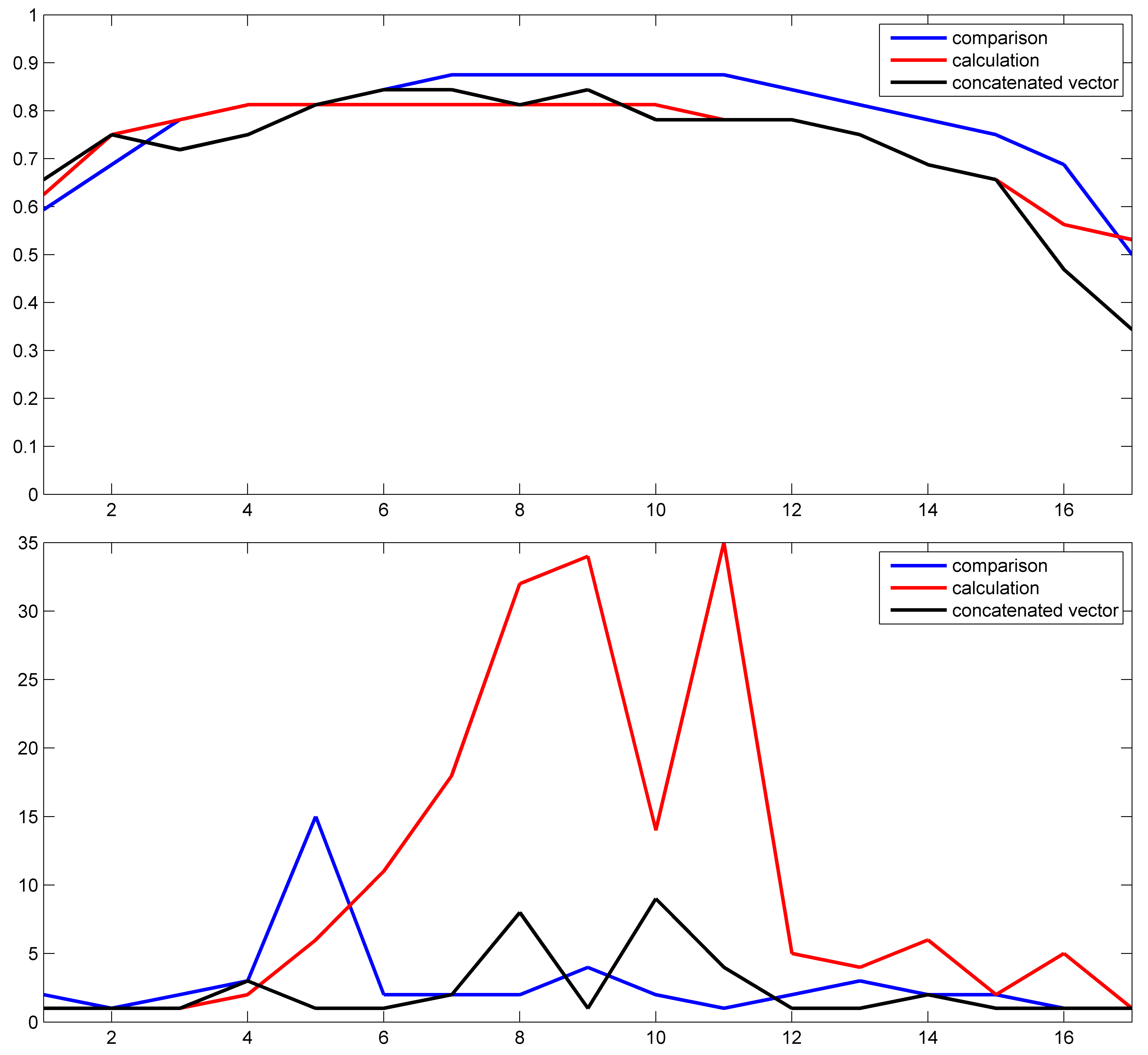

Supplement: Figure S6 — Support-vector machine analysis. This figure depicts the success of the support-vector machine analysis. Top: best correct classification rate (y-axis) for each number of ROIs (x-axis) for each condition. Bottom: Number of different combinations of ROIs (y-axis) that reached the best correct classification rate for each number of ROIs (y-axis). Blue: comparison task, red: calculation task, black: concatenated vector. (TIF) [file pone.0083722.s006.tif]
