# Supplementary material for: Diagnosing Developmental Dyscalculia on the Basis of Reliable Single Case FMRI Methods: Promises and Limitations
Source: PLoS One. 2013 Dec 9;8(12):e83722. doi: 10.1371/journal.pone.0083722 (PMC3857322; doi:10.1371/journal.pone.0083722)
Supplement: Text S1 — Further analyses concerning reliability in single-subject imaging. a. Impact of block time differences due to the self-paced study design. b. Influence of narrow contrasts in single-subject imaging. (DOCX) [file pone.0083722.s007.docx]

**Text S1**

1. **Impact of block time differences on data quality**

In a previous study we showed that self-paced designs are more reliable when compared to classical block designs of various lengths [1]. But, are individual differences in time spent on the tasks correlated with individual differences in signal quality?

There is a link between the number of time points spent on task and the detectable effect size, given a certain level of temporal signal to noise ratio (TSNR) [2]. It has been shown, that TSNR is closely related to the test-retest reliability at the level of the individual [3,4]. One could argue that children who spent more time on task might show better test-retest reliability [5]. In our opinion individual differences in test-retest reliability have a higher priority than individual differences in TSNR, because robust TSNR is a necessary but not a sufficient requirement for test-retest reliability.

Here we investigate this question by correlating reaction time with first-level test-retest reliability estimates. When the correlation is low, no relation between time spent on task and data quality exists. The latter would imply that self-paced block designs are suitable for child studies. In a first step, we will present the first-level reliability estimates; in a second step, we will correlate those estimates with reaction time.

In our opinion individual differences in signal quality are only of interest when they are correlated with individual differences in test-retest reliability. We estimated the link between reliability estimates obtained at the first level and the length of time spent on task. A very standard way to estimate first-level reliability is by means of the so called Dice or Rombout’s overlap measure.


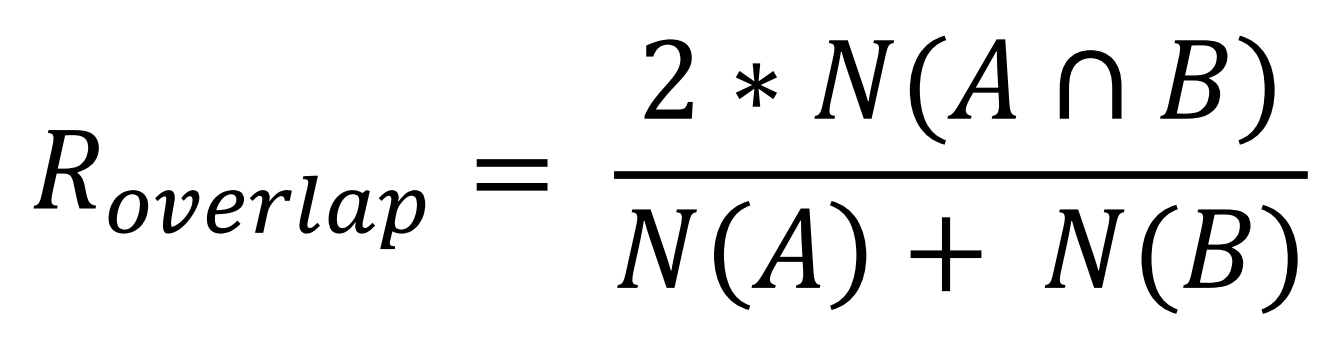


where N equals the number of voxels above the chosen threshold of t-statistics for the test (A) and retest run (B).

However, in our case the use of a t-statistic is not appropriate because the degrees of freedom vary from individual to individual and run to run due to the self-paced design. Hence, we estimated this measure using p-values. The number of detected voxels depends on the chosen threshold, and therefore it affects the Dice overlap measure. The question is how to choose the threshold. Our answer to this question was pragmatic: The reproducibility measure was obtained for each child at all thresholds between 0.05 and 0.00005 in 1000 steps. The results are depicted in Figure S1.

One could of course doubt whether a rough measure like the Dice overlap is in fact a good method to assess the reliability at the level of the individual participant. Alternatively, one could perform a conjunction analysis and afterwards one might correlate the voxels at the level of the individual within the brain areas that survived the conjunction analysis. These reliability maps can be estimated by means of ICC. However for correlations, we extracted the beta weights of the test run and the retest run that lay within the confines of the p-value conjunction analysis. The very large individual differences in reliability have been reported for adults as well and are most likely to be expected [3].

As can be seen in all the graphs in Figure S1, the reliability estimate stays almost constant, irrespective of the chosen threshold. So there is no sizeable difference between first-level reliabilities computed from different thresholds.

In a next step we correlated the reliability estimates of the 32 individual children with the two response times obtained in the test and retest session using a multiple regression approach. It has been advised to apply a Fisher’s z’-transformation to the reliability estimates (Dice overlap & ICC, [3]) before correlating. The resulting curves are depicted in Figure S2.

The multiple correlations between RT and first-level reliability estimates (Dice overlap & ICC) are very low at any of the threshold values. We conclude that there is no relation between time spent on task and data quality.

To a certain extent these results might seem counterintuitive because it has been shown that an increase in time points leads to an increase in signal detection likelihood [2] and reliability [5]. A between task comparison indeed shows that an increase in mean response time or time points leads to an increase in reliability as illustrated by the bold black lines in Figure S1.

How can we explain the poor relation between reliability and number of time points in the experiment? We speculate that slow response times are the consequence of “neural noise” due to inefficient processing. This neural noise is captured in the fMRI signal. In other words the potential increase in signal through an increase in the number of time points is corrupted by the increase in neurally induced noise. As a result, not much of a difference in reliability is observed. It is of course very likely that an increase in the number of stimuli, that inherently goes along with an increase in the number of time points, results in better test-retest reliability. But the absolute number of stimuli seems to be more important than the stimulus density over time.

1. **Baseline contrast vs. narrow contrasts in single-subject imaging**

For clinical investigations of the brain, a sufficient degree of test-retest reliability is essential. But sensitivity and specificity are equally important for accurate description of a disorder. There are many ways to increase specificity of fMRI analysis. For instance one might use narrow task contrasts by employing a control task or one might relate estimates of the ratio or distance effects for number processing to the fMRI signal. In our study, we opted against narrow contrasts because they may have a negative effect on the test-retest reliability or sensitivity.

It is reasonable to assume that the test-retest reliably of the fMRI signal depends on the temporal signal to noise ratio and the effect size of the fMRI contrast computed. It is clear that the smaller the effect size of the contrast the more likely it is that the contrast is dominated by noise [2]. One can compensate for this by increasing the number of observations, but these relations are non-linear in nature. So the number of time points needed for contrasts with small effect sizes increases disproportionately, or alternatively the experiment requires unfeasible TSNR [2].

In the following we will show how the test-retest reliability of the fMRI signal gets corrupted, when narrow contrasts are studied. In a first step we calculated the direct contrast between the comparison and the calculation task. According to Dehaene’s model, the angular gyrus is related to arithmetic fact retrieval [6]. A direct contrast between the non-symbolic calculation task and the non-symbolic number comparison task indeed shows that this region is more activated during arithmetic processing. But it should be mentioned that the up-regulation is found within a region, where the baseline contrast showed a deactivation in both groups. Still, the t-statistics look promising. However, further analyses do not support the quality of these findings.

The test-retest reliability of the non-symbolic calculation task is high with extended areas exhibiting excellent (ICC > 0.75; depicted in yellow), good (ICC > 0.6; depicted in red), and fair (ICC > 0.4; depicted in green) reliability coefficient estimates (Figure S3A and S3B). Moreover the test-retest reliability of the non-symbolic comparison task is very encouraging with values above the fair and good ICC thresholds. In a next step we investigated the test-retest reliability of the narrow contrast non-symbolic calculation – non-symbolic number comparison. This leads to a massive decrease in test-retest reliability. Only very few voxels showed reliability estimates in the good range (Figure S3C). A comparison between the brain activation map and the reliability map shows that the brain activation differences traced are unreliable (Figure S3D). Almost none of them were found within the confines of our very liberal reliability criterion of ICC > 0.33.

Please note that our poor results from narrow contrasts are in line with other studies [3]; they are not due to flaws in the measurements.

In our study we only performed contrasts between the two groups and we did not use a control task in order to calculate narrow contrasts. This would have meant calculating a complex interaction contrast of group and condition. Figure S3 visualizes the effect of one single contrast on the reliability. Calculating a second contrast on the basis of such unreliable data would not lead to valid information.

**References**

1. Krinzinger H, Koten JW, Hennemann J, Schueppen A, Sahr K, et al. (2011) Sensitivity, reproducibility, and reliability of self-paced versus fixed stimulus presentation in an fMRI study on exact, non-symbolic arithmetic in typically developing children aged between 6 and 12 years. Dev Neuropsychol 36 (6): 721–740. doi: 10.1080/87565641.2010.549882.

2. Murphy K, Bodurka J, Bandettini PA (2007) How long to scan? The relationship between fMRI temporal signal to noise ratio and necessary scan duration. Neuroimage 34 (2): 565–574. doi: 10.1016/j.neuroimage.2006.09.032.

3. Raemaekers M, Vink M, Zandbelt B, van Wezel RJA, Kahn RS, et al. (2007) Test-retest reliability of fMRI activation during prosaccades and antisaccades. Neuroimage 36 (3): 532–542. doi: 10.1016/j.neuroimage.2007.03.061.

4. Bennett CM, Miller MB (2010) How reliable are the results from functional magnetic resonance imaging. Ann N Y Acad Sci 1191: 133–155. doi: 10.1111/j.1749-6632.2010.05446.x.

5. Birn RM, Molloy EK, Patriat R, Parker T, Meier TB, et al. (2013) The effect of scan length on the reliability of resting-state fMRI connectivity estimates. Neuroimage 83: 550-558. doi: 10.1016/j.neuroimage.2013.05.099.

6. Dehaene S, Piazza M, Pinel P, Cohen L (2003) Three parietal circuits for number processing. Cogn Neuropsychol 20 (3): 487–506. doi: 10.1080/02643290244000239.
